# The Journal

**Published:** 1854-11

**Authors:** 


### EDITORIAL DEPARTMENT.

### THE JOURNAL.

In commencing the second volume of the Journal, we are encouraged to hope for much in the future by looking back upon the past. We have found, (and we are gratified in announcing the fact;) during the past year, which was the first of its struggling infancy, an ardent desire, on the part of very many of the profession, who have generously aided us by the procurement of subscribers and in contributing to its columns, to sustain us in an undertaking they well knew was unrewarded, save by *that* gratifying consideration alone. It was all we asked, but at the same time we were taught to expect it for various reasons, the first and most prominent of which was, that such a work was *imperatively demanded* by the wants of an intelligent profession around us. Isolation is a condition at which the human mind revolts, for we look with pitying and tearful eye upon the self-exiled recluse. A profession isolated and separated from its kindred, is in no condition to advance but is rather retrograding, it matters not how much have been their previous acquirements. There is no *statu quo* state of the mind, it must advance or if not, it must recede. If even the profession of a state or district alone were to adopt measures for self-advancement, it would not, with the most untiring efforts, be able to take rapid strides in advancement, but the members must lag behind the great body of the profession of the world around them, and with whom they hold no intercourse. But unfortunately for such hopes, the truth is that not only a local profession, without an organ or vehicle of communication of its own, is shut out from the medical world, but at the same time the members composing it are individually shut away from one another. Medical men must deeply feel the embarrassment of their situation when they reflect that there are others engaged in the same active and responsible duties, in a country of the same or similar medical topography, daily in a conflict with diseases of the same type, compelled to acknowledge features of these in obscurity and defying successful explanation, and yet at the same time there is nothing known of the experience and success of their neighbors in this perpetual strife with the maladies incident to the country, for want of that medium of introduction, thought, and intercommunication. None other of the learned professions could subsist as such, or prove useful in their vocations, without the means of ready and constant communication. In the progress of society exigencies arise which require the application of new rules to meet or to overcome, and he of the "black cloth" is required to expose some new *ism* found current, but heterodox in the world around him and which threatens to advance with its destroying influences into the midst of his flock and scatter them away from the fold. Or, perhaps some new scheme of moral and religious improvement has been projected in a distant community, highly favorable to moral reformation, and yet if he adopted a monkish seclusion, those who leaned upon him for instruction would be denied the benefits of the march of progress. Our relations with each other are daily changing and with these changes new rules are demanded. The statesman will look for data in the experience of those among whom these changes first began, as a rule of his action. So also with the legal profession, new conditions of society impose new obligations and the relations of property and persons are thereby often modified. New difficulties spring up and new causes of difference arise between members of society. Modifications of the rules of law are demanded and new decisions are had in accordance with the rights of persons in these new relations. The judiciary then look beyond their own benches for criteria by which to rule their own judgements and decisions. So also in the medical profession. As new luxuries are introduced, new customs adopted, or different climacteral influences at war with the continued preservation of health, new forms of disease will arise or if not old and familiar types will be changed and so modified as to present an almost entirely new phaze. The sparsely settled portions of the country may for a long time remain exempt, and yet a denser human settlement will bring with it these legitimate consequences. We might refer to instances strongly in point. The cholera was unknown in this country until 1832, and yet through the columns of the Medical Journals its progress, symptoms and fatality, from Bengal through a devious course until it finally, at the above date, reached Paris and London, and very soon after appearing at Quebec on the 8th of June of that year, it spread with rapidity through the entire continent. But its symptoms were so well known that it was soon recognised, nor was there allowed much time for speculation, for so rapid was its spread, and such the mortality which followed it, that no one doubted but that the angel of death was surely among us. Its habitudes were known and our people were somewhat prepared for its reception. We know not how much more fatal that visitation would have proved had it not been for the adoption of the most rigid sanatory and dietetic observances, with other precautionary measures. Not so when it visited China nor Hindostan. The former in their high assumptions and celestial arrogance, had previously shut themselves up and denied all intercourse with the world, and hence they knew nothing of the pathology, treatment or prevention of the disease and hence too its fatality among a people crowded together, poorly fed, and filthily inclined. We refer to these instances, not with the view of entering upon a desertation upon Cholera, but to show the necessity of putting ourselves in an intimate relation with the medical world. In this particular the Journal places the profession in that relation, for like the lens in an optical instrument, it embraces a wide field and concentrates the objects so that they may be seen at one view, or like the mirror reflecting each object faithfully and truly. The time was when the medical literature of the country rested for its diffusion upon the journals of the Eastern Atlantic cities, but now they are found in all sections of the country, radiating their light to the profession around them. When collected together they constitute as a whole, a valuable record and furnish a fund of sedate and valuable material for scientific advancement. Doubtless they have their defects, but of this it is not our province to speak now, that duty will be discharged at its appropriate time and in its proper place.

But it is not to be expected that a single medical man can avail himself of the benefits of all these contributions to the medical science of the country, nor would he have the leisure, apart from his duties, to examine and cull out from the mass, what would more particularly interest him in his particular locality. Laborious as the task is, we have undertaken to reflect, among our brethren, the light which from every cardinal point of the country, it is our good fortune, through the friendly agency of our Journal, to have thrown in upon us.

These journals have come to us recently many of them contain-taining the most flattering encomiums upon the proud position which the profession of our young State now occupies in that of the country. Even the British Continental Journal at Montreal has been pleased to bestow the mead of high praise. Will our medical men continue to deserve it? Will they stifle emulation and not seek for a still higher position by a still higher merit? Are they disposed to permit the elements of improvement and advancement to struggle for diffusion or wither and die for want of a material basis? Is there professional *State pride* enough to preserve and increase the capacities of their *only* organ or medium of communication? We have an abiding faith that they will not, but that they will take their Journal in their hands and with proud exultation claim it *as their own*, and vow that it shall continue theirs. Let them exhibit it as a true evidence of their devotion to medical science and as a proof that they stand prominent in their profession. Let its pages show that they have contributed to its columns as confirmation of their position and that their labors are appreciated.

To one and all "*to whom these presents may come*" we say forward your names, but yet be not satisfied that you have then done your *whole* duty. By no means—it does not rest there.—You will call upon your neighbors and by appeals to their good sense, their generosity and professional pride, induce them to enrol themselves upon the list among our subscribers. In taking up that list we can point with exultation to quite a long one and because their names are *there*, we regard as *prima facia* evidence of their prominent position at home, among those for whom they labor, and the profession generally.

The first number then of the second volume of our Journal is now before you. We rely in confidence upon your liberality, and justice in criticism, and that it may be so far approved and judged worthy as to make it an acceptable *bi-monthly* visitant. Remember this the *only work of the kind in the State*, and be assured too that so long as we receive the encouragement which has been extended to it during the first year of its existence, we will not only continue it, but that in proportion as it receives *substantial* approbation it will be still further improved and amended. As an inducement and incentive we will present to that individual at the close of the current volume, a work on medicine which he will regard as worthy of his effort, in obtaining the greatest number of good subscribers.

